# Retraction notice for: Therapeutic effect and potential mechanism of pioglitazone in rats with severe acute pancreatitis. [Braz J Med Biol Res (2018) 51(2): e6812]

**DOI:** 10.1590/1414-431X20186812retraction

**Published:** 2018-05-21

Wang Hai^1,2^, Xu Ping^2,3^, Yang Zhi-wen^2,3^ and Zhang Chun^2^

^1^Department of Shanghai East Hospital, Tongji University, Shanghai, China

^2^Shanghai Songjiang Hospital, Nanjing Medical University, Nanjing, China

^3^Department of Gastroenterology, Songjiang Hospital Affiliated Shanghai First People’s Hospital, Shanghai Jiao Tong University, Shanghai, China

**Retraction for:** Braz J Med Biol Res | doi: 10.1590/1414-431X20176812 | PMID: 5731332

The authors would like to retract the article “Therapeutic effect and potential mechanism of pioglitazone in rats with severe acute pancreatitis” that was published in volume 51 no. 2 (2018) (Epub Dec 11, 2017) in the *Brazilian Journal of Medical and Biological Research* <http://dx.doi.org/10.1590/1414-431x20176812> PMID: 5731332.

The first author Wang Hai states that “we want to do further study about the part of validation of NF-κB, p38MAPK proteins”.

The Brazilian Journal of Medical and Biological Research had received authorization from all authors before the publication of the paper. We regret the unprofessional behavior of the authors involved.

